# Development of a Practical Model to Predict Conversion to Total Hip Arthroplasty Following Non-Vascularized Bone Grafting

**DOI:** 10.3389/fsurg.2022.835128

**Published:** 2022-04-01

**Authors:** Shihua Gao, Liang Mo, Kaishen Cai, Wei He, Ziqi Li

**Affiliations:** ^1^ Guangzhou University of Chinese Medicine, Guangzhou, China; ^2^ Traumatology and Orthopedics Institute, Guangzhou University of Chinese Medicine, Guangzhou, China; ^3^ The Third Affiliated Hospital, Guangzhou University of Chinese Medicine, Guangzhou, China

**Keywords:** osteonecrosis of the femoral head, non-vascularized bone grafting, total hip arthroplasty, nomogram, prediction model

## Abstract

**Objective:**

To identify risk factors of failure after Non-Vascularized Bone Grafting (NVBG) in osteonecrosis patients, establish and validate a nomogram predictive model of hip survival after NVBG.

**Methods:**

Data on ONFH patients undergoing NVBG at our institution between 2010 and 2017 were retrospectively collected. Preoperative risk factors potentially associated with failure after NVBG were assessed by univariate Cox regression analyses. A predictive nomogram was developed based on multivariate Cox regression model. The performance of the nomogram model was evaluated by C statistic. Subjects were stratified according to total points calculated from the nomogram and Kaplan-Meier curves were plotted to further evaluate the discrimination of the model. The model was also internally validated through calibration curves.

**Results:**

The overall 2-year and 5-year hip survival percentages were 91.8 and 84.6%, respectively. Age, etiology, Association Research Circulation Osseous stage and range of necrotic lesion were independent risk factors of failure after NVBG. The C statistic of the nomogram model established with these predictors was 0.77 and Kaplan-Meier curves of the tertiles showed satisfactory discrimination of the model. Internal validation by calibration curves indicated favorable consistency between actual and predicted hip survival rate.

**Conclusion:**

This predictive model may be a practical tool for patient selection of NVBG. However, future studies are still needed to externally validate this model.

## Introduction

Osteonecrosis of the femoral head (ONFH) is a disabling disease and patients often end up with arthroplasty (1). Although total hip arthroplasty (THA) has been proven to be an effective intervention for osteonecrosis of the femoral head (ONFH) (2), the risk of revision procedures could not be ignored, especially the patients of ONFH are mostly adolescent and young adults, for whom the goal of treatment should be to delay or even avoid THA. Non-vascularized bone grafting (NVBG) is a classic method of hip preserving surgery, but the reported success rate varied between 55 and 87% (3–5). However, there is still no consensus on the indication of NVBG for the treatment of ONFH, and patient selection criteria of previous studies differ from one another. It is possible that the uncertainty of success rate was associated with patient selection criteria, because blindly expanding the scope of application of NVBG in the treatment of ONFH could lead to early failure. Therefore, it is important to further clarified the patient selection criteria of NVBG.

Few studies have specifically investigated risk factors of failure after NVBG and there is also no predictive model to assist surgeons or patients in making their clinical decisions. Nomogram is a visualization method to present a predictive model, which have been widely used in prognostic researches of oncology. Developing a nomogram predictive model based on disease related risk factors can present the prognostic information of each patient in a more straightforward way. In addition, a nomogram model can be used to accurately stratify patients, and therefore it could be used as a tool to assess whether an individual patient could benefit from a certain treatment method (6).

In this study, our purposes are to identify risk factors of conversion to THA after NVBG in osteonecrosis patients and to establish and validate a practical preoperative assessment model for prediction of hip survival after NVBG to assist in making clinical decisions.

## Patients and Methods

### Inclusion and Exclusion Criteria

Our study was conducted in accordance with the “Transparent Reporting of a multivariable prediction model for Individual Prognosis or Diagnosis (TRIPOD)” (7). Data were collected from a retrospective database consisting of 250 ONFH patients with 299 hips treated with hip preservation surgery at a single center between 2010 and 2017. Only patients who underwent non-vascularized surgery were included in the current study. Patients with prior hip preservation surgeries because of ONFH were excluded. Patients with missing values on predictors or those under 16 years old were not included in the analysis. Informed consents were obtained from all patients and this study were approved by the hospital ethics committee.

### Surgical Procedure

When using the Phemister technique to implement a fibular graft, the patient was placed on the operating table in a semi-lateral position (operating side tilted upwards by 15°–20°). After locating the necrotic lesion of the femoral head, a 6 to 8 cm lateral incision was made to expose the grater trochanter of the femur, and a 2 mm Kirschner wire (K-wire) was inserted through the lateral of subtrochanteric area. The tip of the inserted K-wire should be advanced to the subchondral bone of the femoral head, which was around 5 mm from the joint line on anteroposterior (AP) and lateral views. Guided by the inserted K-wire, the reamer for the dynamic hip screw was used to drill a bone tunnel with a diameter that can match a fibular allograft. Through the bone tunnel, necrotic lesion was gently removed using a bone curette. Caution must be made to avoid penetrating the cartilage of the femoral head. Then, impaction grafting was performed with a mixture of viable cancellous bone harvested during the preparation of the bone tunnel and allogenic bone chips to filled the cavity in the femoral head. After measuring the depth of the remaining bone tunnel, the fibular bone graft with proper length was inserted into the tunnel. Finally, the fibular graft was fixed by a titanium screw with appropriate length.

We used the Smith-Petersen approach to perform the lightbulb and trapdoor procedure. The patient was placed on the table in a supine position with the operating hip slightly elevated using pads. An anterolateral incision of approximately 15 cm was made, after dividing the subcutaneous fat tissue, the gap between the tensor fasciae latae and sartorius was dissected. Then, the gluteus medius was retracted laterally and the rectus as well as the iliopsoas were retracted medially. Through the plane between gluteus medius and rectus femoris, the capsule of the hip was exposed. A T-shape incision was made on the anterior portion of the capsule and the femoral head was exposed. In the lightbulb procedure, an approximately 2 cm × 2 cm cortical window was created in the femoral head-neck junction using a thin osteotome. In the trapdoor procedure, a chondral window was made on the femoral head surface. Through either of these two windows, debridement of necrotic lesion was carried out using a burr with a mushroom like tip. All of the necrotic bone was removed until exposure of bleeding viable bone surface. Then, full thickness iliac crest with appropriate size and cancellous bone chips were harvested on the anterior third of iliac crest. The harvested iliac crest was used as strut graft and autogenous cancellous bone chips were packed tightly into the void in the femoral head. Finally, the cortical or osteochondral flap was replaced, the femoral head was reduced and the joint capsule was repaired.

All patient received education about rehabilitation exercise including gradually increase hip range of motion and isometric contraction of quadriceps. Weight-bearing was not allowed for the operated side for at least three months after surgery. If signs of bone repair in the femoral head were shown on plain radiographs after three months, patients were allowed to progress to partial weight-bearing. Patients were followed up every three months after the surgery for the first year, then every six months for another two years, and annually thereafter. AP and frog lateral (FL) plain radiographs were taken at each postoperative follow-up appointment to evaluate the healing of necrotic lesion and progression of osteoarthritis. Follow-up would be ended when patients receive THA or having a plan to receive THA within a month.

### Recorded Data

Baseline characteristics and conversion to THA were derived from medical records and postoperative follow-ups. Demographic and clinical data were collected, including age, gender, body mass index (BMI), laterality (affected side and whether contralateral hip was affected), etiology (traumatic vs non-traumatic), duration of pain (≤6 months vs >6 months), classification of ONFH, approach of bone grafting and duration of follow-up. The dependent variable was conversion to THA or having a plan to receive THA within a month.

Each hip diagnosed with ONFH was classified utilizing the 2019 version of Association Research Circulation Osseous (ARCO) staging system (8) and Japanese Osteonecrosis Investigation Committee (JIC) (9) classification based on preoperative AP plain radiographs and MRI. We also classified included hips based on frog leg lateral plain radiographs using the similar criteria as the original JIC classification. Included hips were further classified according to the range of necrotic lesion, which was succinctly represented by whether the lesion in the femoral head exceeded the edge of acetabulum on both AP and FL views. There were four types of ONFH according to this classification criteria (Table 1). All radiographic evaluation was completed by an experienced orthopedic surgeon blinded to the medical records and outcomes of the subjects.

**Table 1:** Classification criteria based on range of necrotic lesion.

Types	Over the edge of acetabulum on AP view	Over the edge of acetabulum on FL view
A1F1	−	−
A2F1	+	−
A1F2	−	+
A2F2	+	+

*AP, anteroposterior; FL, frog lateral.*

### Statistical Analysis

Continuous variables were reported as medians and interquartile ranges (IQRs), and categorical variables were reported as number and proportions. Probability of survival and median follow-up time of the operated hips were generated using the Kaplan-Meier method. Difference in survival was examined by the log-rank test. The association between hip survival and variables of clinical importance or published in previous studies (10–14) were assessed by univariate Cox regression analyses.

Backward step wise selection with Akaike information criterion (AIC) was applied to identify variables in the final multivariate Cox regression model. Then, selected variables of the final model were used to established a nomogram to predict the probability of 2-year and 5-year survival after non-vascularized bone grafting. The discrimination performance of the nomogram was validated by Harrell’s C statistics. A C statistic of 1.0 indicates the model have perfect discrimination, while a C statistic of 0.5 indicates the model has no discrimination (15). Kaplan-Meier curves were plotted over the tertiles of subjects stratified by the total points calculated from the nomogram. Finally, calibration performance was evaluated by calibration curves with bootstrapped resampling of the study group. All tests in this study were two sided, and *P* value ≤0.05 was considered statistically significant. All statistical analyses were performed with statistical software (R, version 4.0.5; http://www.r-project.org).

## Results

### Baseline Characteristics

A total of 299 hips were identified, among which 51 hips were excluded because preoperative radiographs were unable to retrieve or the affected hip had undergone other hip preservation surgery previously, leaving 248 hips from 215 patients for final analyses.

**Figure 1:**
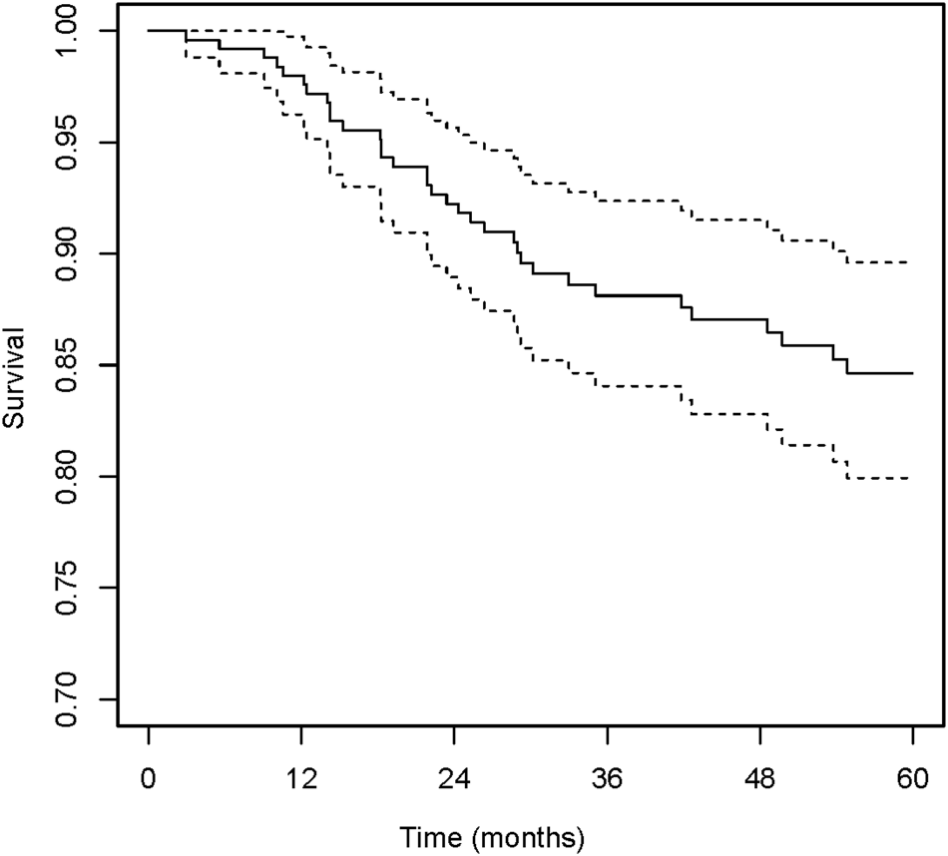
The Kaplan-Meier survival curve of the entire cohort.

The median age at the time of surgery was 33.5 years (IQR, 16–59 years), and the median of BMI was 24.6 kg/m^2^ (IQR, 22.7–25.3 kg/m^2^). Most of the hips were from male patients (72.2% [179 of 248]) and bilateral ONFH (71.8% [178 of 248]). The etiology of ONFH was primarily non-traumatic (88.3% [219 of 248]). In total, 79.2% [168 of 235] of the hips were in pain with a duration of 6 months or shorter. All of the included hips were at ARCO grade II, IIIA and IIIB assessed from preoperative radiographs, and the percentages were 37.1% (92 of 248), 42.7% (106 of 248) and 20.2% (50 of 248), respectively. On AP views, the percentages of type C1 and C2 were 31.0% (77 of 248) and 60.1% (149 of 248) respectively, while only 8.9% (22 of 248) of the hips were JIC type B. The distribution was similar classified from FL views, 7.3% (18 of 248) of the hips were type B, and the percentages of type C1 and C2 were 39.9% (99 of 248) and 52.8% (131of 248), respectively. When classified based on the range of necrotic lesion, 29.0% (72 of 248) were type A1F1, 18.2% (45 of 248) were type A2F1, 10.9% (27 of 248) were type A1F2 and 41.9% (104 of 248) were type A2F2. There were 151 hips (60.9%) underwent bone grafting in the Phemister approach, 58 hips (23.4%) in the lightbulb approach and 39 (15.7%) in the trapdoor approach. The median of duration of follow-up was 79.3 months (IQR, 3.0–132.9 months). Details of baseline characteristics were shown on Table 2. In the whole study group, during a median follow-up time of 71.03 months (95% confidence interval [CI], 64.97–74.93), the overall incidence of THA conversion was 17.3% (43 of 248). The 2-year and 5-year hip survival percentages were 91.8% (95% CI, 88.5–95.3%) and 84.6% (95% CI, 79.9–89.6%), respectively (Figure 1).

**Table 2:** Baseline characteristics of the hips undergoing non-vascularized bone grafting.

Characteristic	Total (*n* = 248)
Age, yr, median (IQR)	33.5 (27–41)
BMI, kg/m^2^ (IQR)	24.6 (22.7–25.3)
Male	179 (72.2%)
Left	142 (57.3%)
Bilateral	178 (71.8%)
Etiology	
Traumatic	29 (11.7%)
Non-traumatic	219 (88.3%)
Duration of pain (*n* = 235)	
≤6 months	186 (79.2%)
>6 months	49 (20.8%)
ARCO	
II	92 (37.1%)
IIIA	106 (42.7%)
IIIB	50 (20.2%)
JIC on AP	
B	22 (8.9%)
C1	77 (31.0%)
C2	149 (60.1%)
JIC on FL	
B	18 (7.3%)
C1	99 (39.9%)
C2	131 (52.8%)
Range of necrotic lesion	
A1F1	72 (29.0%)
A2F1	45 (18.2%)
A1F2	27 (10.9%)
A2F2	104 (41.9%)
Approach of bone grafting	
Phemister	151 (60.9%)
Lightbulb	58 (23.4%)
Trapdoor	39 (15.7%)

*IQR, interquartile range; BMI, body mass index; ARCO, Association Research Circulation Osseous; JIC, Japanese Osteonecrosis Investigation Committee; AP, anteroposterior; FL, frog lateral.*

### Risk Factors Associated with Conversion to THA

Results from univariate Cox regression showed that age (hazard ratio [HR], 1.05; 95% CI, 1.02–1.09), ARCO grade IIIB (HR, 3.66; 95% CI, 1.60–8.40), type A2F2 range of necrotic lesion (HR, 3.83; 95% CI, 1.58–9.29), bilateral ONFH (HR, 2.57; 95% CI, 1.08–6.09) and undergoing the lightbulb approach (HR, 1.96; 95% CI, 0.99–3.88) may be potential risk factors for conversion to THA after non-vascularized bone grafting surgeries. Characteristics of clinical importance or previously published risk factors were selected as candidate predictors of the model. Using the AIC backward stepwise selection identified four variables in multivariate Cox regression modeling, including age, etiology, ARCO staging and range of necrotic lesion. According to multivariate analysis, age (HR, 1.07; 95% CI, 1.04–1.11), non-traumatic ONFH (HR, 7.31; 95% CI, 0.99–53.95), ARCO grade IIIB (HR, 4.19; 95% CI, 1.68–10.48) and type A2F2 range of necrotic lesion (HR, 3.77; 95% CI, 1.49–9.51) were each independently associated with conversion to THA. Details were shown on Table 3.

**Table 3:** Cox proportional hazards regression model showing the correlation of factors with survival.

Variables	Univariable		Multivariable	
	HR (95% CI)	*P* value	HR (95% CI)	*P* value
**Factors selected**				
Age	1.05 (1.02, 1.09)	<0.01	1.07 (1.04, 1.11)	<0.001
Etiology				
Traumatic	1 [Reference]	\	1 [Reference]	\
Non- traumatic	5.16 (0.71, 37.52)	0.11	7.31 (0.99, 53.95)	0.05
ARCO				
II	1 [Reference]	\	1 [Reference]	\
IIIA	2.00 (0.91, 4.43)	0.09	1.78 (0.78, 4.05)	0.17
IIIB	3.66 (1.60, 8.40)	<0.01	4.19 (1.68, 10.48)	<0.01
Range of necrotic lesion				
A1F1	1 [Reference]	\	1 [Reference]	\
A2F1	2.22 (0.71, 6.92)	0.17	1.65 (0.51, 5.30)	0.22
A1F2	1.99 (0.56, 7.06)	0.29	2.23 (0.62, 7.94)	0.40
A2F2	3.83 (1.58, 9.29)	<0.01	3.77 (1.49, 9.51)	<0.01
**Factors not selected**				
BMI	1.14	0.37	\	\
Duration of pain				
≤6 months	1 [Reference]	\	\	\
>6 months	0.79 (0.33, 1.90)	0.60	\	\
Bilateral				
No	1 [Reference]	\	\	\
Yes	2.57 (1.08, 6.09)	<0.05	\	\
Approach of bone grafting				
Phemister	1 [Reference]	\	\	\
Lightbulb	1.96 (0.99, 3.88)	0.05	\	\
Trapdoor	1.76 (0.80, 3.91)	0.16	\	\

*IQR, interquartile range; ARCO, Association Research Circulation Osseous; JIC, Japanese Osteonecrosis Investigation Committee; AP, anteroposterior; FL, frog lateral.*

### Nomogram and Model Performance

**Figure 2:**
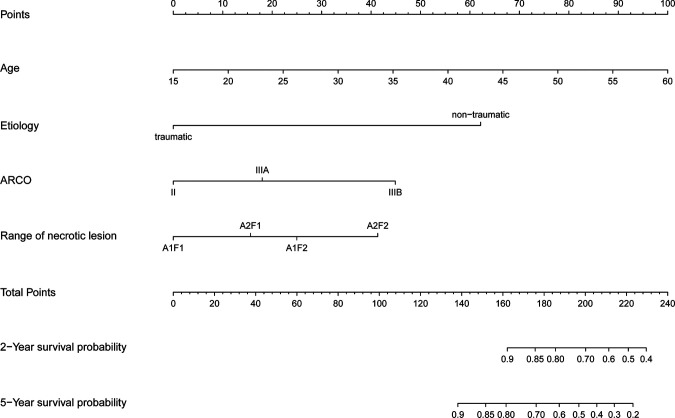
The nomogram to predict 2-year and 5-year hip survival was established based on four independent prognostic factors.

Nomogram to predict survival of the ONFH hips after non-vascularized bone grafting is shown in Figure 2. The nomogram was established based on the above mentioned four predictors. For each predictor, the length of the line segment corresponds to the number of points. A higher sum of assigned points was associated with higher risk of conversion to THA. Also, according to the total points, the 2-year and 5-year hip survival rate can be obtained from the nomogram.

**Figure 3:**
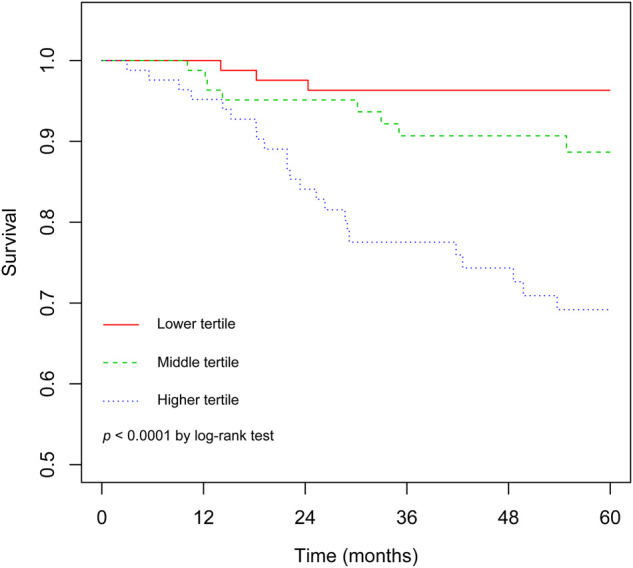
The Kaplan-Meier curves demonstrating survival of hips following non-vascularized bone grafting according to tertiles of predicted points.

**Figure 4:**
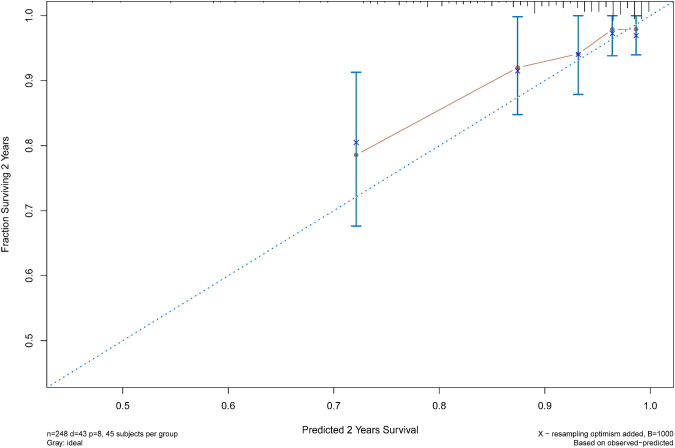
The calibration curves for evaluation of the consistency between the actual and the predicted hip survival rates. Satisfactory consistencies were showed on 2-year survival probability.

**FIGURE 5:**
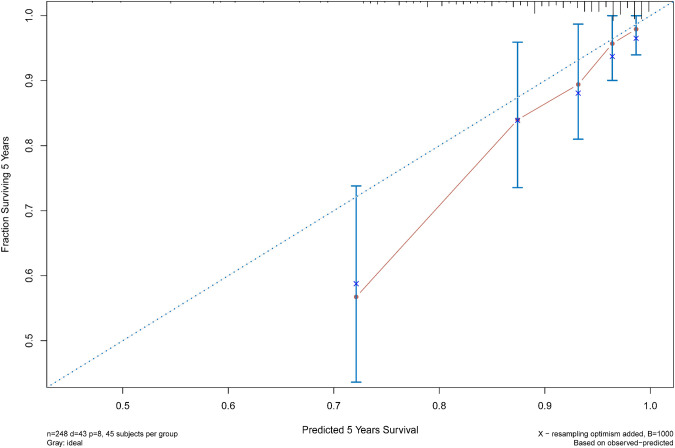
The calibration curves for evaluation of the consistency between the actual and the predicted hip survival rates. Satisfactory consistencies were showed on 5-year survival probability.

The C statistic of the model was 0.77 (95% CI, 0.69–0.84), indicating the discrimination of the current model was favorable. Kaplan-Meier curves of hip survival stratified by tertiles of total points calculated from nomogram further showed the discriminative ability of the model (Figure 3). Log-rank test showed significant difference of hip survival among tertiles (*p *< 0.0001). The model was then evaluated for accuracy and potential overfitting by plotting bootstrapped calibration curves (45-sample, 1,000 resamples), and the consistency between actual and predicted hip survival probabilities for both time points was acceptable. Calibration curves for 2-year and 5-year are shown in Figures 4 and 5.

## Discussion

Predicting conversion to THA after NVBG is necessary. In our study, we identified preoperative risk factors of conversion to THA following NVBG. Based on the selected risk factors, we established a practical model to predict the prognosis of patients undergoing NVBG for the first time. With this tool, surgeons could identify patients at high risk of early conversion to THA in a clearer way.

In our model, age is a relatively dominant factor in predicting conversion to THA. Previous studies have reported that patients with younger age at the time of receiving the hip preservation procedures would have better outcomes. They evaluated patients undergoing the Phemister approach of NVBG and the results demonstrated patients under 30 years have higher chance of clinical success (10, 14). The result from the study of Rijnen et al. also implicated that NVBG could be an effective hip preservation technique even in a late stage for very young patients (10). On the other hand, it is reported that ONFH induced by corticosteroid use may have poorer prognosis (10, 11). In our analysis, non-traumatic ONFH was a significant and independent risk factor for conversion to THA after NVBG. Zhang et al. reported a study on a group of 28 teenage patients with traumatic ONFH undergoing free vascularized fibular grafting and the outcome was encouraging, with none of them required conversion to THA during follow-up, suggesting hip preservation surgeries may be more beneficiary for young patients with traumatic ONFH (16).

Plenty of studies have pointed out that the preoperative stage of ONFH is an independent risk factor of failure following NVBG (11, 13, 17). Multivariate Cox analysis of our data also showed ARCO stage was an important risk factor in predicting failure after NVBG. We considered the extent of preoperative collapse could reflect the difficulty of restoring the sphericity and mechanical strength of femoral head and therefore preoperative staging could be a predictor of prognosis.

The range or location of the necrotic lesion is also an important factor that should be taken into account in the treatment of ONFH (18). Seyler et al. reported outcomes of 39 hips underwent NVBG with a mean of 36 months follow-up. Their results showed that failure (defined as not converting to THA) after NVBG was correlated with the location of lesion, with 71% of JIC type C2 failed while the incidences were 17 and 42% for type C1 and type B, respectively. Therefore, necrotic lesion exceeding the lateral edge of acetabulum could be an indicator of failure after NVBG. On the other hand, anterior involvement of the femoral head should also be taken into consideration. Previous studies on hip joint mechanics have indicated that the stress is greatest on the anterolateral femoral head, and the peak stress can be 4 to 7 times body weight with daily activities such as walking, sitting, and squatting (19–21). Compared to computed tomography or magnetic resonance imaging, evaluating anterior involvement on FL radiograph view may be a more economic and practical method (22). A recent study has pointed out the importance of evaluating the anterior portion of necrotic lesion on the FL view, with more anterior lesions yielding poorer outcomes (23). In our study, we tried to depict the range of necrotic lesion based on whether the lesion exceeded the edge of acetabulum on both AP and FL views (Table 1), which turns out to be a relatively comprehensive and practical way of assessing the range of necrotic lesion.

The exact indication of NVBG is hard to define because the prognosis after NVBG is co-affected by multiple risk factors. Hence, we established a data driven predictive model and visualized it as a nomogram so that we could easily evaluate the prognosis of individual patient preoperatively. Based on this model, we could optimize the indication of NVBG by selecting patients with lower possibilities to failed after the surgery. Moreover, patients could have a more straightforward understanding of their prognosis after NVBG and participate in clinical decision efficiently.

The present study had limitations. First of all, the clinical result of this group of patients may only reflect the performance of our institute, which may compromise the generalizability of our model. In addition, the retrospective nature of this study may induce potential bias even though data was collected from preexisting medical records and blinding method was used in the process of radiographic evaluation. Finally, because there was no available data from other hospitals, external validation was not performed. Despite internal validation showed satisfactory performance of the nomogram model, external validation was still necessary to further confirm the reliability of the current model.

## Conclusion

In conclusion, from the data set of ONFH patients undergoing NVBG, several important predictors of short-term and mid-term hip survival were identified, including age, etiology, ARCO stage and range of necrotic lesion. Using these prognostic predictors, the nomogram of the current study was able to stratified ONFH hips into different prognostic groups regarding the risk of conversion to THA and internal validation showed satisfactory discrimination and calibration performances. This predictive model may be a potential tool for patient selection of NVBG. However, future studies are still needed to externally validate this model.

## Data Availability

The raw data supporting the findings of this study are available from the authors upon reasonable request.
